# Headaches in the emergency department –a survey of patients’ characteristics, facts and needs

**DOI:** 10.1186/s10194-019-1053-5

**Published:** 2019-11-05

**Authors:** Alberto Doretti, Irina Shestaritc, Daniela Ungaro, John-Ih Lee, Loukas Lymperopoulos, Lili Kokoti, Martina Guglielmetti, Dimos Dimitrios Mitsikostas, Christian Lampl, on behalf of the School of Advanced Studies of the European Headache Federation (EHF-SAS)

**Affiliations:** 1 Department of Neurology-Stroke Unit and Laboratory of Neuroscience, Istituto Auxologico Italiano, Milan, Italy; 2 Department of Neurorehabilitation, Yaroslavl regional clinical hospital of War Veterans, Yaroslavl, Russian Federation; 3 Department of Neurology, Medical Faculty, Heinrich-Heine-University, Duesseldorf, Germany; 4 80th Battalion of Medical Corps, General Ioannis Makrygiannis Camp, Pyli, Kos, Greece; 5 General Hospital of Grevena, Grevena, Greece; 6 Regional Referral Headache Centre, Sant’ Andrea Hospital, Rome, Italy; 7 Department of Medical, Surgical and Experimental Sciences, University of Sassari, Rome, Italy; 8 1st Neurology Department, Aeginition Hospital, Medical School, National and Kapodistrian University of Athens, Athens, Greece; 9 Headache Medical Center Linz, Ordensklinikum Barmherzige Schwestern, Seilerstaette 2-4, 4020 Linz, Austria

**Keywords:** Headache, Emergency department, Primary headache, Migraine, Secondary headache

## Abstract

**Background and aim:**

Headache is very often the cause for seeking an emergency department (ED). However, less is known about the different diagnosis of headache disorders in the ED, their management and treatment. The aim of this survey is to analyse the management of headache patients in two different ED in Europe.

**Methods:**

This retrospective survey was performed from September 2018 until January 2019. Patients were collected from the San Luca Hospital, Milan, Italy and the Ordensklinikum Barmherzige Schwestern, Linz, Austria. Only patients with a non-traumatic headache, as the primary reason for medical clarification, were included. Patients were analysed for their complexity and range of examination, their diagnoses, acute treatment and overall efficacy rate.

**Results:**

The survey consists of 415 patients, with a mean age of 43.32 (SD ±17.72); 65% were female. Technical investigation was performed in 57.8% of patients. For acute treatment non-steroidal-anti-inflammatory drugs (NSAIDs) were the most used, whereas triptans were not given. A primary headache disorder was diagnosed in 45.3% of patients, being migraine the most common, but in 32% of cases the diagnosis was not further specified. Life-threatening secondary headaches accounted for less than 2% of cases.

**Conclusions:**

The vast majority of patients attending an ED because of headache are suffering from a primary headache disorder. Life-threatening secondary headaches are rare but seek attention. NSAIDs are by far the most common drugs for treating headaches in the ED, but not triptans.

## Introduction

Headaches are one of the most challenging complaints in the emergency department (ED) accounting for 1–4% of all ED visits [1–4]. Headache types, diagnostic procedures and acute treatment may vary in different EDs throughout countries, depending on the catchment area, specific departments of the hospital, structure of their particular ED, in-house protocols and local medical staff. In addition, clinicians in the ED are busy, usually with a limited time setting and in general facing two challenges: filtering patients who need further diagnostic evaluation, including neuroimaging and lumbar puncture in specific cases, as well as the aim of headache relief by adequate treatment. Life-threatening conditions presenting with headache embrace cerebrovascular, brain mass effect and inflammatory-infection pathologies mainly, yet rare compared to primary headache disorders. To screen patients for a plausible secondary headache in ED, physicians should consider the suggested “red flag” symptoms in patients presenting with headache [5]. Red flag symptoms are numerous and do not include exclusively neurological signs. Moreover, potential co-morbidities, specific headache history and individualised patient characteristics should be considered. In a few studies exploring the frequency of secondary headaches in ED, approximately 5% of patients with severe headaches had a secondary headache [6], some of them life-threatening or severe disabling [7]. However, the majority of patients had a benign diagnosis. Published data about headache patients in the ED, their diagnoses and management are rare, especially when comparing different countries.

In this current study we provide results on characteristics and management of non-trauma headache patients in the ED of two different European Union cities. These results were compared with the so far published surveys and critically discussed.

## Methods

This cross-sectional cohort study was performed retrospectively from September 2018 until January 2019. Patients were collected from two hospitals in Europe: the San Luca Hospital of Istituto Auxologico Italiano, Milan, Italy and the Ordensklinikum Barmherzige Schwestern, Linz, Austria.

The San Luca Hospital in Milan is a Scientific Institute for Hospitalisation and Care including a non-profit organisation for biomedical and high specialization hospital treatment. This ED consists of 3 different examination rooms. A cardiologist, nurses and other paramedics are in charge and a neurologist on call. In 2018, 11.073 patients visited the ED and the physicians examined 923 cases (mean) per month.

The Ordensklinikum Barmherzige Schwester is a general hospital in Linz with 663 beds and 17 different departments and institutes. In the ED general practitioners, internal medicine physicians, neurologists and nurses are working in an interdisciplinary setting. The hospital has 8 specific admission days per month. In 2018, 26.978 patients visited the ED and the physicians examined 207 cases (mean) per month.

The study conformed to the revised ethical principles of the Helsinki declaration and the Codex rules and guidelines for research. It is based on patients who participated in the inpatient assessment and treatment program in the both ED. During their first appointment all patients participating in the survey provided written informed consent to use their data for the quality control, and to publish the data in anonymized form as part of the quality control process. Therefore, an ethics approval was not obtained for the present analysis.

Patients were eligible for inclusion if they presented to the ED with an acute or longer lasting, disabling non-traumatic headache of any potential cause. The headaches were characterized with descriptive statistics calculated for patient demographic, clinical findings, investigations, and ED diagnosis. As analyses were considered exploratory, no formal adjustment for multiple comparisons was performed. In an exploratory analysis, both hospitals were compared, using the chi-square test, Fisher’s exact test, Student t-test, or Wilcoxon rank-sum test as appropriate. Statistical significance was set at a < 0.05 (two-sided). Patients were analysed for their complexity and range of examination, their diagnoses, acute treatment intervention and overall efficacy rate at discharge (Patients Global Impression (PGI) Q:” Have you been satisfied with the examination and treatment regime - Yes/No?”).

## Results

### Demographics

The survey consists of 415 patients (184 patients from the San Luca Hospital and 231 patients from the Ordensklinikum Barmherzige Schwestern, Linz). 268 (65%) were female and 147 (35%) male, with a mean age of 43.32 (SD ±17.72; ranged from 15 to 96 years). Overall, non-traumatic headache accounted for 3.2% of the total ED visits (3.5% in Linz and 2.9% in Milan), being disproportionately more females (65% vs. 35%, *p* = .001). Eighty-two of all patients (19.8%) presented vomiting as a concomitant symptom. Five patients (2.7%) had a history of non-headache specific trauma in their history (Table 1).

**Table 1:** Demographics and patients characteristics

	Both hospitals *n* = 415 (%)	Linz, Austria *n* = 231 (%)	Milan, Italy *n* = 184 (%)	*p*-value
Age (y), M, SD,	43.32 ± 17.72	42.37 ± 17.74	51.26 ± 16.46	<.001
(range)	(15–96)	(15–96)	(15–85)	
Female	268 (65.0)	141 (39.0)	126 (68.5)	.000
Male	147 (35.0)	90 (61.0)	58 (31.5)	.000
Mean time since onset (in hours)		20.71 ± 88.31 (range 0–1095 h)	175.56 ± 476.72 (range 0.33–4120 h)	.000
Neurological Exam	301 (72.5)	197 (85.3)	104 (56.5)	.000
Technical investigation				
Head CT	221 (53.2)	109 (47.2)	112 (60.96)	.007
Brain MRI	7 (1.7)	4 (1.7)	3 (1.6)	.445
Angio-CT	12 (2.9)	4 (1.7)	8 (4.3)	.144
Lumbar puncture	5 (1.2)	2 (0.9)	3 (1.6)	.659
Facial TC Scan	6 (1.4)	0 (0)	6 (1.6)	
EEG	1 (0.2)	0 (0)	1 (0.3)	
Cervical RX	1 (0.2)	0 (0)	1 (0.3)	
Other examination				
Blood test	333 (80.0)	163 (58.2)	170 (46.1)	
ECG	225 (54.0)	42 (15.0)	183 (49.7)	
ENT examination	5 (1.2)	5 (1.8)	0 (0)	
Ophthalmological examination	4 (1.0)	4 (1.2)	0 (0)	

*ECG* Electrocardiogram, *ENT* Ear-Nose-Throat, *CT* Computer Tomography, *MRI* Magnet

### Work-up and treatment

Neurological examination was performed in 301 of patients (72.5%), statistically significant more in Linz than in Milan ((197, 85.3% vs 104; 56.5%, *p* = .000). Technical investigation was performed in 253 patients (60.9%); 221 patients (53.2%) had a non-contrast cranial CT, 12 patients (2.9%) received a cranial CT with additional CT angiography and 7 patients (1.7%) underwent a brain MRI. A lumbar puncture was performed in five patients (1.2%). Thirty-five patients (8.4%) were admitted to the local neurological department for further investigations, because they had focal neurological symptoms. Other examinations consist of blood test (*n* = 333; 80%), ECG (*n* = 225; 54%), ENT and opthalmological examination were done in 9 patients (2.2%).

For acute treatment (n=408) non-steroidal-anti-inflammatory drugs were given in 237 patients (58.0%), acetaminophen in 58 patients (14.2%), 2 patients (0.5%) received corticosteroids and 94 (23.0%) received other drugs (including aspirin, antiemetics, metamizol, paracetamol). Triptans were not given in any patients. Further therepeutic recommendations at discharge were shown in Table 2. The overall efficacy rate (PGI) was satisfactory in 195 patients (47.4%). A therapeutic recommendation at discharge was given in 401 patients (96.6%).

**Table 2:** Therapeutic procedures

	Acute therapy in the ED			Therapeutic recommendations at discharge		
	All *n* = 408 (%)	Linz, Austria *n* = 227 (%)	Milan, Italy *n* = 181 (%)	All *n* = 401 (%)	Linz, Austria *n* = 231 (%)	Milan, Italy *n* = 170 (%)
NSAIDs	237 (58.0)	142 (62.5)	95 (52.5)	164 (40.9)	102 (44.1)	62 (36,5)
Steroids	2 (0.5)	1 (0.4)	1 (0.5)	0 (0)	0 (0)	0 (0)
Triptans	0 (0)	0 (0)	0 (0)	9 (2.2)	3 (1.3)	6 (3.5)
Acetaminophen	58 (14.2)	18 (7.9)	40 (22.1)	50 (12.5)	28 (12.1)	22 (12.9)
Others	94 (23.0)	53 (23.3)	41 (22.7)	155 (38.7)	75 (32.5)	80 (4.7)
missing data	17 (4.1)	17 (7.4)	0 (0)	23 (5.7)	0 (0)	23 (13.5)

*ED* Emergency department, *NSAIDs* Non-steroidal anti-inflammatory drugs, *N/A* Non applicable

### Diagnosis

The final diagnoses, according to the ICHD 3 classification of headache disorders [8] are shown in Table 3. A primary headache was found in 188 patients (45.3%), while 90 patients (21.7%) had a secondary headache, a trigeminal neuralgia was diagnosed in 4 patients (1.0%). Headache diagnosis was not further specified in 133 patients (32.0%).

**Table 3:** Final diagnosis at discharge

Type of headache	All Hospitals *n* = 415 (%)	Linz *n* = 231(%)	Milan *n* = 184(%)	X^2^	*p*-value
Primary Headache					
Unknown cause					
(„not better specified“)	133 (32.0)	92 (42.4)	41 (22.2)	14.47	<.001
Migraine	110 (26.5)	59 (25,5)	61 (33,2)	2.886	.102
TTH	68 (16.4)	44 (19.0)	24 (13.0)	2.695	.110
TTH + migraine	10 (2.4)	10 (2.4)	0 (0)	8.162	.003
TACs	0 (0)	0 (0)	0 (0)		
Secondary Headache					
Sinusitis	18 (14.3)	15 (6.5)	3 (1.6)	5.838	.016
Hypertension	33 (7.95)	12 (5.2)	21 (11.4)	5.410	.027
SAH	5 (1.2)	1 (0.4)	4 (2.2)	2.608	.176
Meningitis or encephalitis	1 (2.4)	0 (0)	1 (0.3)	1.258	.443
Neuralgia					
Trigeminal neuralgia	4 (0.96)	2 (0.9)	2 (1.1)	.052	1

*TTH* Tension-type headache, *TACs* Trigeminal autonomic cephalalgias, *SAH* Subarachnoid haemorrhage

287 (69.1%) were discharged at home, 84 (20.2%) has to be transferred to the ED short-stay unit for further observation. Thirty-five patients (8.4%) were admitted to the neurological department for additional investigation.

## Discussion

In our current analysis of patients visited the ED because of headache, in both hospitals migraine has been reported to be the most common cause (26%) of primary headaches. Similar migraine diagnosis rate in the EDs was found in a cross-sectional Australian study [10]. In contrast to that, a recent published, large epidemiological US trial showed over a 10-year period of observation, that migraine was present in 63.5% of all headache presentations in an ED [7]. One possible explanation for this major difference comes up from the American Migraine Study published in 1998: Lipton and colleagues estimated that only 66% of migraine sufferers had ever consulted a doctor for headache [9], and one of the major causes for this is an insufficient outpatient care and/or low consultation rates. Therefore more headache sufferers make a claim on ED to seek help for their burden. Other major findings, as the predominance of female headache patients in the ED, are in line with previous observations in the United States of America [6], in Australia [10], as well as in Brazil [11]. Compared to lower percentages (14.5% or 38%) found in previous studies [4, 10] in our survey 53% patients received CT scans in the ED.

Vomiting was a frequent observation in headache ED patients, which is explained by the fact that vomiting is one of the cardinal symptoms of migraine, and migraine is the most common cause of primary headaches in the ED [4, 10]. Likewise, another study has reported a higher percentage of 31.7% [10]. The percentage of life threatening secondary headache diagnoses (bleedings, meningitis/encephalitis) in our study was below 2%, which is also in agreement (< 3%) with previous studies published [4, 10].

About 32% of the headaches in this study were simply labelled as “headache” without a more specific ICHD 3 diagnosis [8]. This is lower than the 44% reported by Chu and colleagues [10] and similar to the 36% reported by Friedman and colleagues [12], who conducted detailed structured patient interviews with the assistance of trained research associates. However, the ratio of headache cases not further specified remains high in our survey, outside the expected limits. A possible reason may be related to the multi-professional staffing of the two hospitals. General practitioners, internal medicine physicians, cardiologists, and neurologists work together to provide a multidisciplinary approach for these patients; therefore not all headache patients were seen and managed by neurologists, or by a headache specialist. Furthermore, the physicians must be able to address the patient’s need for pain management in a very short and intense timeframe and exclude simultaneously any possibility of a life threatening illness. The challenge in the busy setting of an ED is to decide within a limited amount of time, which patient is in need of immediate further diagnostic work up in order to exclude any secondary, dangerous headaches, that might have serious and irreversible health consequences, if diagnosis and treatment are delayed.

Surprisingly, 94% of our patients received non-specific simple analgesic drugs for acute headache therapy, but none received a triptan, although they were available in both ED. For patients presenting de novo to an ED for management of migraine, emergency clinicians clearly have a broad armamentarium of therapeutic options. In these cases, we speculate emergency clinicians might choose to use non-specific simple analgesic drugs instead of triptans for one of the following reasons: (i) they believe that non-specific simple analgesic drugs are effective and appropriate for the acute treatment of migraine; (ii) they are concerned about adverse events of triptans; (iii) they are not sufficiently familiar with triptans; (iv) they prefer a treatment parentally administrated for faster efficacy and/or because of vomiting (only sumatriptan is available in this formulation); (iv) ED were not supplied with any triptan. However, triptans were prescribed in 9 patients (2%) at discharge. Reasons for the low application of triptans should be investigated in further studies. This could be of special interest for care managers, because previously published data revealed that patients who received triptans had the shortest median length of stay in the ED [13].

In our setting 53% of patients had a CT head scan, which is very much higher than in other surveys [5, 10, 11]. Reasons for that may be complex. Physicians often work under the pressure of time constraints, and initial assessment, including SNNOOPS 10 list [14] can be difficult, especially in patients with pre-existing neurological or psychological conditions. Studies have reported difficulties in making a definite headache diagnosis in the setting of ED [15–17] without any CT scan of the brain. However, a careful history and physical examination remain the most important parts of the assessment of the headache patients in order to identify high-risk patients and to exclude any secondary headache, that, if left without treatment, could have disastrous effects on the patient’s health [7, 10]. Patients who have one or more high-risk historical features or examination findings are considered to have a life-threatening condition requiring urgent diagnostic work-up [18, 19]. Red flag symptoms include neurological signs or symptoms (confusion, seizure, altered mental status, loss of consciousness, asymmetric reflexes, focal neurologic deficits or visual deficits), meningism, fever, sudden and severe onset of the headache or change in the characteristics of a known headache, advanced age (onset after 50 to 65 years), pregnancy or puerperium, coagulopathy, neoplasm history, positional headache, headache precipitated by sneezing, coughing or exercise, painful-eye with autonomic features, posttraumatic onset of headache, painkiller overuse or new drug at onset of headache and any systemic disease including HIV infection and any immunosuppressed state in general [6, 10, 14]. The European Headache Federation consensus reports the reasons and headache cases that may need technical investigation, as well as the required tests [5].

### Study limitations and strengths

There are several limitations that should be addressed. First, the study was a retrospective data analysis and all clinical data were collected by the treating physician and not by dedicated trained headache experts. Second, the treating physicians did not record data. Eligibility was not verified, nor was missing cases specifically sought. Systematic selection bias is possible, but unlikely given cases were enrolled in the ED 24 h per day by many clinicians across both sites. For the data collection, questions on clinical history were probably different in both centres, as there was no uniform and standardised questionnaire. Whether missing data may bias the results will depend on whether the data were missing at random or not. The latter could be problematic. On the other hand, this is the first survey conducted in Europe reporting real word data for the management of headache in an ED setting. This information may trigger relevant organisations and health care designers to improve care delivered in ED.

## Conclusion

Patients with non-traumatic headache as the primary presenting symptom in the ED are more often women than men. The majority of headache patients in the ED had primary headaches with migraine being the most frequent diagnosis. Life threatening secondary headaches, including SAH and meningitis/encephalitis, were rare, accounting for less than 2% of the patients. NSAIDs and acetaminophen were the most commonly used symptomatic therapy of headaches, while triptans were not used in the ED.

## Data Availability

The datasets analysed during the current study are available from the corresponding author on reasonable request.

## Abbreviations

CT: Computer Tomography
ECG: Electrocardiogram
ED: Emergency Department
ENT: Ear-Nose-Throat
ICHD III: International Classification of Headache Disorders, 3rd edition
MRI: Magnet Resonance Imaging
N/A: Non applicable
NSAIDs: Non-steroidal-anti-inflammatory-Drugs
PGI: Patients Global Impression,
SAH: Subarachnoid haemorrhage
TACs: Trigeminal autonomic cephalalgias
TTH: Tension-type headache
